# Acute Upper Gastro-Intestinal Bleeding in Morocco: What Have Changed?

**DOI:** 10.5402/2011/457946

**Published:** 2011-07-24

**Authors:** A. Timraz, W. Khannoussi, F. Z. Ajana, W. Essamri, I. Benelbarhdadi, R. Afifi, M. Benazzouz, A. Essaid

**Affiliations:** Medical Unit C, Ibn Sina Hospital, Rabat, Morocco

## Abstract

*Objective*. In the present study, we aimed to investigate epidemiological, clinical, and etiological characteristics of acute upper gastro-intestinal bleeding. *Materials and Methods*. This retrospective study was conducted between January 2003 and December 2008. It concerned all cases of acute upper gastroduodenal bleeding benefited from an urgent gastro-intestinal endoscopy in our department in Morocco. Characteristics of patients were evaluated in terms of age, gender, medical history, presenting symptoms, results of rectal and clinical examinations, and endoscopy findings. *Results*. 1389 cases were registered. As 66% of the patients were male, 34% were female. Mean age was 49. 12% of patients had a history of previous hemorrhage, and 26% had a history of NSAID and aspirin use. Endoscopy was performed in 96%. The gastroduodenal ulcer was the main etiology in 38%, followed by gastritis and duodenitis in 32.5%. *Conclusion*. AUGIB is still a frequent pathology, threatening patients' life. NSAID and aspirin are still the major risk factors. Their impact due to peptic ulcer remains stable in our country.

## 1. Introduction

Acute upper gastro-intestinal bleeding (AUGIB) is a very common medical emergency. It is associated with high mortality rates despite recent developments in diagnosis and treatment methods [1, 2]. Peptic ulcer and portal hypertension are the two main causes [1–3]. In the present study, we aimed to investigate epidemiological, clinical, and etiological characteristics of AUGIB and to compare our results with those of the same endoscopy unit.

## 2. Materials and Methods

This retrospective study was conducted in the Medical Unit C in Ibn Sina hospital in Morocco, between January 2003 and December 2008. It concerned all cases of AUGIB having underwent an urgent gastro-intestinal endoscopy in our department. Firstly, patients' management was in the emergency department for setting condition and possible reanimation and stabilization of their homodynamic status. The patients were then transferred to our department for endoscopy. After, they returned to the emergency department or their original unit with results. Characteristics of the patients were evaluated in terms of age, gender, medical history, presenting symptoms, results of rectal and clinical examinations, and endoscopy findings.

## 3. Results

**Figure 1:**
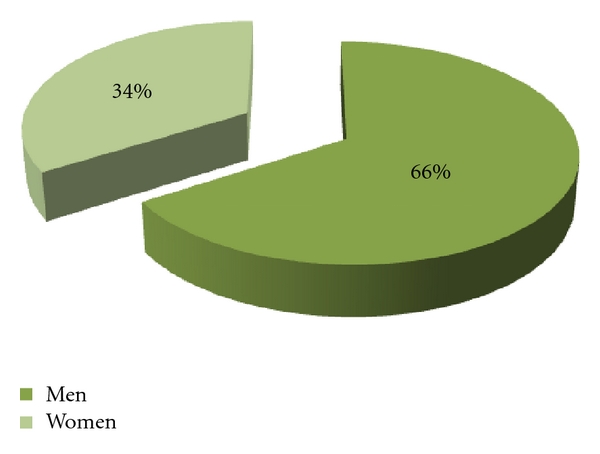
Repartition of patients by gender.

During this period (2003 to 2008), 1389 who presented AUGIB were enrolled in this study. As 66% of the patients were male, 34% were female with a sex ratio of 2.2 (Figure 1).

Mean age was 49. The youngest was an adolescent of 12 years old who presented esophageal varices stage III, and the oldest was a woman of 100 years old who presented esophagitis stage III.

 Three hundred and fifty-eight cases (26%) were enrolled in 2003 and 121 (9%) in 2004, 237 (17%) in 2005, 322 (23%) in 2006, 182 (13%) in 2007, and 169 (12%) in 2008 (Table 1).

While hemorrhage was determined for the first time in 1219 (88%) of cases, 170 (12%) had a history of previous hemorrhage. 362 patients (26%) had used one or more gastrotoxic drugs (aspirin, NSAID, corticosteroids). The medical history revealed gastric and duodenal ulcers in 13%, and varices in 16%. Alcohol history was present among 1% of patients, and cigarette smoking was present among 10% of them (Table 2). 

**Figure 2:**
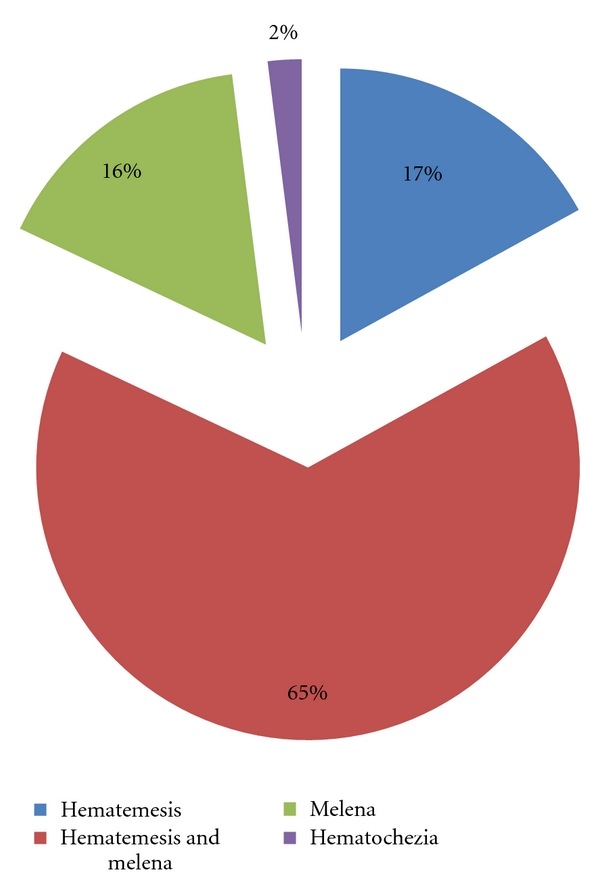
Nature of the hemorrhage.

While 239 (17%) cases described hematemesis, 224 (16%) patients described melena and 897 (65%) described both hematemesis and melena. 29 patients (2%) had one or more episodes of important hematochezia requiring the use of an urgent endoscopy (Figure 2).

Hemorrhagic shock was found in 133 patients (9.5%). 65 patients (4.7%) presented disorders of consciousness. Rectal examination showed melena in 1028 patients (74%) and hematochezia in 34 patients (2.5%) (Table 3).

Nine hundred and twenty-four patients of 1015 (93.4%) had anemia with hemoglobin less than 10 g/dL. 157/625 patients (25%) had thrombocytopenia (Table 4).

The most common finding among endoscopy results was gastroduodenal ulcer (38%) with 25% for duodenal ulcer and 13% for gastric ulcer. In 453 cases (32.5%), it was gastritis and duodenitis. 326 patients (23.5%) had an AUGIB by rupture of esophageal varices. In 56 patients (4%), the endoscopy was strictly normal (Table 5).

## 4. Discussion

AUGIB is one of the most common emergency conditions associated with digestive system, and it exhibits significant morbidity and mortality rates [4]. Their early management is capital because it can reduce mortality, recurrence, and duration of hospitalization of patients [5, 6]. Upper digestive endoscopy is a capital time to precise bleeding source. If bleeding is not controlled, endoscopy must be done urgently as soon as security conditions are met. If bleeding is controlled, it can be deferred for several hours to perform the act in the best conditions [7]. Currently, there is an overall decrease in the incidence of AUGIB which remains highly variable from one study to another [8]. In our series the incidence has decreased from 26% in 2003 to 12% in 2008.

The incidence of AUGIB has been reported to be 60–70%, higher among males [8]. In the current study, parallel with the literature, 66% of our patients were male. Our population has a mean age of 49 years with extremes ranging from 12 to 100 years. These data coincide with those described in the two series in the same endoscopic unit in 1982 and 1999 [9, 10], but they are different from those described in the literature, where the mean age was between 58 and 73 years [5, 8, 11–13]. This can be explained by the youth of the Moroccan population. 

Age is considered by most authors as a capital factor in the occurrence of AUGIB. They can appear more frequently in the elderly population [13]. In the present study, 12% of patients had a history of a previous bleeding. Fiore et al. reported the rate of patients with a previous bleeding as 19–23% [15]. Recent studies report a decreasing trend in this number [16]. Compared to those studies, the rate found in our study was observed to be low. 

Many studies have shown a relationship between NSAID and aspirin use, and AUGIB [8, 17]. Gallerani et al. have confirmed that NSAID compared with other drugs were associated with the highest risk of hospitalization for AUGIB [18]. Another study conducted in Spain had shown an increasing number of deaths by AUGIB in patients treated with NSAIDs [8]. Corticosteroids do not appear to increase the risk of ulcer bleeding unless they are used in combination with NSAIDs [19].

 Fiore et al. reported the rate of aspirin use in 1996 and 2000 as 27% and 33%, respectively [15]. Recent studies demonstrate those rates to be around 40–65% [16]. In the current study, the rate of NSAID and aspirin use was 26%. This is higher than the last series of the same endoscopic unit in 1999 (5%) [9]; this can be explained by the automedication more and more frequent in Moroccan population. The risk of AUGIB related to the antiplatelet drugs has been well established. It is especially shown in patients aged >70 years [20]. The clinician is still facing the problem: stopping antiplatelet with the risk of thromboembolic complications or continuing with the risk of further bleeding. The Standards of Practice Committee of the American Society of Gastro-Intestinal Endoscopy (ASGE) suggests that patients with AUGIB taking antiplatelet agents should have these medications withheld until hemostasis is achieved. Administration of platelets may be appropriate for patients with life-threatening or serious bleeding. They recommend that patients with AUGIB receiving anticoagulant therapy have these agents withheld until hemostasis is achieved [21].

As well as drug use, alcohol and smoking habits have an important place among risk factors of AUGIB [22]. Fiore et al. found alcohol habit in 70% of cases having AUGIH [15]. In the present study, the rates of alcohol and smoking were 1% and 10%, respectively. 

 AUGIB appears in 75% of cases by hematemesis associated or not with melena, in 20% of cases by melena and in less than 5% of cases by hematochezia showing evidence of a very active postpyloric bleeding [4]. These data coincide with our series. 

The most common underlying cause of AUGIH is peptic ulcer [15]. Studies show that peptic ulcers are the underlying reason in 45–60% of patients across the world who present with AUGIB [15, 16].

Currently, many recent studies tend to show a decreasing of peptic ulcer disease and its bleeding complications [8]. These studies did not show any decrease in the frequency of the rupture of esophageal varices [8]. Comparing our endoscopic data with four previous endoscopic series run in the same endoscopic unit (Table 6) shows that the ulcer disease remains the main etiology of AUGIB despite the wide spread of inhibitors proton pump, while gastritis and duodenitis came in second place before the rupture of esophageal varices, which can be explained by the increasing use of gastrotoxic drugs. The AUGIB by rupture of esophageal varices increased comparing to previous series; this was explained by the increasing incidence of chronic hepatitis and probably by the better management of portal hypertension. In different series of the literature, peptic ulcer remains the main cause of AUGIB, followed by the rupture of esophageal varices [5, 8, 12, 14] (Table 7).

## 5. Conclusion

Acute upper gastro-intestinal bleeding remains a frequent pathology, threatening patients' life. It mainly affects men. Using gastrotoxic drugs increases the risk of these AUGIB Peptic ulcer causing AUGIB remains stable in our country.

## Figures and Tables

**Table 1:** Distribution of AUGIB by year.

Year	Number	%
2003	358	26
2004	121	9
2005	237	17
2006	322	23
2007	182	13
2008	169	12

**Table 2:** Medical history.

	Number	%
Previous hemorrhage	170	12
Varices	221	16
Gastroduodenal ulcers	181	13
Drugs	362	26
Alcohol	14	1
Cigarettes	136	10

**Table 3:** Clinical data.

	Number	%
Hemorrhagic shock	133	9.5
Consciousness disorders	65	4.7
Pallor	1052	76
Melena	1028	74
Hematochezia	34	2.5

**Table 4:** Biological data.

	Number	%
Anemia (Hb <10 g/dL)	924/1015	91
Hyperleucocytosis	256/614	41
Thrombopenia	157/625	25
Thrombocytosis	37/625	6

**Table 5:** Endoscopy findings in patients.

	Number	%
Gastroduodenal ulcer	530	38
Gastritis and duodenitis	453	32.5
Esophageal varices	326	23.5
Esophagitis	312	22.5
Cardial varices	108	8
Gastric tumor	50	3.6
Hypertensive gastropathy	41	3
Mallory Weiss	15	1.1
Esophageal tumor	10	0.7
Gastric polyp	8	0.5
Dieulafoy ulcer	4	0.3
Angiodysplasia	4	0.3
Duodenal tumor	3	0.2
Hemobilia	2	0.07

**Table 6:** Evolution of causes of AUGIB in our department [9].

Year	1982	1991	1999	2006	Our series
Number	460	1062	600	600	1389
Ulcer disease	40%	39%	39%	37%	38%
Esophageal varices	18%	15%	22%	22%	23%
Gastritis and duodenitis	16%	15%	16%	16%	32%

**Table 7:** Causes of AUGIB (comparison with the literature).

Author	Number	Ulcer disease	Gastritis and duodenitis	Esophageal varices
Tammaro et al. (Italy) [5]	436	50%	18%	12%
Kasem et al. (England) [12]	121	29%	21%	3%
Dursun et al. (Turkish) [14]	1242	34%	14%	31%
Essaid (Morocco) [1]	1389	38%	32%	23%
